# Factors Associated With Return to Work After Acute Myocardial Infarction in China

**DOI:** 10.1001/jamanetworkopen.2018.4831

**Published:** 2018-11-21

**Authors:** Zihan Jiang, Rachel P. Dreyer, John A. Spertus, Frederick A. Masoudi, Jing Li, Xin Zheng, Xi Li, Chaoqun Wu, Xueke Bai, Shuang Hu, Yun Wang, Harlan M. Krumholz, Hong Chen

**Affiliations:** 1 Health Care and International Medical Services, Peking Union Medical College Hospital, Beijing, People’s Republic of China; 2 Department of Emergency Medicine, Yale School of Medicine, New Haven, Connecticut; 3 Center for Outcomes Research and Evaluation, Yale–New Haven Hospital, New Haven, Connecticut; 4 Department of Biomedical and Health Informatics, University of Missouri–Kansas City; 5 Department of Cardiovascular Research, St Luke’s Mid America Heart Institute, Kansas City, Missouri; 6 Department of Medicine, University of Colorado School of Medicine at the Anschutz Medical Campus, Aurora; 7 Colorado Cardiovascular Outcomes Research Consortium, Denver; 8 National Clinical Research Center of Cardiovascular Diseases, State Key Laboratory of Cardiovascular Disease, Fuwai Hospital, National Center for Cardiovascular Diseases, Chinese Academy of Medical Sciences and Peking Union Medical College, Beijing, People’s Republic of China; 9 Department of Biostatistics, Harvard T.H. Chan School of Public Health, Boston, Massachusetts; 10 Section of Cardiovascular Medicine, Department of Internal Medicine, Yale School of Medicine, New Haven, Connecticut; 11 Department of Health Policy and Management, Yale School of Public Health, New Haven, Connecticut; 12 Department of Cardiology, Peking University People’s Hospital, Beijing Key Laboratory of Early Prediction and Intervention of Acute Myocardial Infarction, Beijing, People’s Republic of China

## Abstract

**Importance:**

Return to work is an important indicator of recovery after acute myocardial infarction. Little is known, however, about the rate of returning to work within the year after an acute myocardial infarction in China, as well as the factors associated with returning to work after an acute myocardial infarction.

**Objectives:**

To determine the rate of return to work within 12 months after acute myocardial infarction, classify the reasons why patients did not return to work, and identify patient factors associated with returning to work.

**Design, Setting, and Participants:**

This prospective cohort study, conducted in 53 hospitals across 21 provinces in China, identified 1566 patients who were employed at the time of the index acute myocardial infarction hospitalization and participating in the China Patient-centered Evaluative Assessment of Cardiac Events Prospective Study of Acute Myocardial Infarction. Data collected included patients’ baseline characteristics; employment status at 12 months after acute myocardial infarction; and, for those who were not employed at 12 months, potential reasons for not returning to work. A logistic regression model was fitted to identify factors associated with returning to work at 12 months. Data were collected from January 1, 2013, through July 17, 2014, and statistical analysis was conducted from August 9, 2016, to August 15, 2018.

**Main Outcomes and Measures:**

Return to work, defined as rejoining the workforce within 12 months after discharge from hospitalization for the index acute myocardial infarction.

**Results:**

Of 1566 patients (130 women and 1436 men; mean [SD] age, 52.2 [9.7] years), 875 patients (55.9%; 95% CI, 53.4%-58.3%) returned to work by 12 months after acute myocardial infarction. Among the 691 patients who did not return to work, 287 (41.5%) were unable to work and/or preferred not to work because of acute myocardial infarction and 131 (19.0%) retired early owing to the acute myocardial infarction. Female sex (relative risk, 0.65; 95% CI, 0.41-0.88), a history of smoking (relative risk, 0.82; 95% CI, 0.65-0.98), and in-hospital complications during the index acute myocardial infarction (relative risk, 0.96; 95% CI, 0.93-0.99) were associated with a lower likelihood of returning to work.

**Conclusions and Relevance:**

Almost half of the previously employed Chinese patients did not return to work within 12 months after acute myocardial infarction. Female sex, history of smoking, and in-hospital complications were associated with a lower likelihood of returning to work.

**Trial Registration:**

ClinicalTrials.gov Identifier: NCT01624909

## Key Points

**Question:**

What patient factors are associated with return to work within 12 months after myocardial infarction in China?

**Findings:**

In this cohort study of data from the China Patient-centered Evaluative Assessment of Cardiac Events Prospective Study of Acute Myocardial Infarction conducted in 53 hospitals across 21 provinces in China, of 1566 patients who were employed at the time of the index hospitalization, only 55.9% returned to work by 12 months after acute myocardial infarction. Women were less likely to return to work, and smoking status and in-hospital complications were the only preventable factors that were associated with a lower likelihood of returning to work.

**Meaning:**

Reducing in-hospital complications and promoting smoking cessation may be important targets for interventions focused on a timely return to the workforce after acute myocardial infarction.

## Teaser

This cohort study determines the rate of return to work among Chinese patients within 12 months after acute myocardial infarction, classifies the reasons why patients did not return to work, and identifies patient factors associated with returning to work after acute myocardial infarction.

## Introduction

Return to work after acute myocardial infarction (AMI) is an important indicator of recovery. It signals a transition from illness to an active social position and has been associated with physical and psychological well-being,^1,2^ life satisfaction,^3^ and quality of life.^4^ Failure to return to work may increase the incidence of depression,^5,6,7^ decrease quality of life,^7^ increase the financial burden on patients and their families,^8^ and impose societal costs, such as worsening labor shortages.^9^ Return to work after AMI is particularly important in China, where patients presenting with AMI are younger than in many other countries.^10^ Moreover, because of the impending increase in the retirement age to 65 years for many jobs in China, return to work will become a relevant measure of recovery for even more people.

Return to work after AMI involves a combination of physical and psychological functions (able to return), sociodemographic factors (must return), and affinity for work (want to return). Previous studies have shown that returning to work is directly influenced by the recurrence of coronary heart disease,^11^ psychological function,^1,11,12^ and socioeconomic factors.^13,14,15^ However, most of these studies were cross-sectional or retrospective, based on relatively small and single-center study samples, and conducted more than a decade ago. To date, to our knowledge, no specific study about returning to work after AMI has been conducted in China, and the proportion of the Chinese population with AMI who return to work and the factors associated with this decision remain largely unknown. It is also unclear whether patients’ reported reasons for not returning to work are associated with their physical health, mental health, or sociodemographic characteristics.

Accordingly, we used data from the China Patient-centered Evaluative Assessment of Cardiac Events Prospective Study of Acute Myocardial Infarction (China PEACE Prospective Study of AMI)—one of the largest and most contemporary samples of working-age patients hospitalized for AMI for a study conducted in 53 hospitals across 21 provinces in Chine^16^—to determine the rate of return to work within 12 months after AMI, classify the reasons why patients did not return to work, and identify patient factors associated with returning to work. We hypothesized that return to work would be associated with patient sociodemographic and medical characteristics. Our goal was to generate evidence that supports improvements in clinical practice and outcomes, including higher rates of returning to work after AMI in China.

## Methods

### Study Sample and Data Collection

The China PEACE Prospective Study of AMI consecutively enrolled 5901 patients with AMI aged 18 years or older who were discharged alive from acute care hospitals in China from January 1, 2013, through July 17, 2014. Patient information was collected during the index hospitalization for AMI and after discharge at 1, 6, and 12 months.^16^ Medical record data were centrally abstracted for the index hospitalization, and interview data were collected at baseline (ie, index hospitalization) and during follow-up at 1, 6, and 12 months after discharge. Real Data Medical Research Inc, Ningbo, China, abstracted all medical records. All follow-up interviews were conducted either in person or via telephone. After discharge, patients were instructed to return to the hospital for follow-up interviews with site investigators. Telephone interviews were conducted only when in-person interviews were not feasible. Patients were classified as nonresponders after 5 telephone interview attempts with no response. The overall response rate was 97.8% (4118 of 4212). The study was approved by the ethics committee of Fuwai Hospital, the national coordinating center for China PEACE, and, where required, by the local ethics committees of individual hospitals. Patients provided written informed consent. This study followed the Strengthening the Reporting of Observational Studies in Epidemiology (STROBE) reporting guidelines.

For the current study, we restricted the sample to 1662 patients who reported having a job (part-time or full-time) at the time of the index AMI hospitalization, agreed to participate in the follow-up interviews, were discharged alive and not transferred to another acute care hospital, and completed the 12-month interview. Because patients who did not return to work after the index AMI may have retired, we excluded 96 employed patients who had reached retirement age (for most occupations, ≥55 years for women and ≥60 years for men) by the 12-month interview. This restriction did not apply to self-employed patients.

### Outcome

Our outcome was return to work, defined as full-time or part-time employment at 12 months after discharge from the index hospitalization for AMI. Among patients who did not return to work within 12 months, we assessed the following reasons for not returning: (1) not returning to work owing to AMI (if a patient reported being unable to work or preferring not to work because of AMI), (2) early retirement owing to AMI (if a patient reported retiring after the index AMI but had not yet reached retirement age), (3) full-time homemaker or unemployed (if a patient reported becoming a full-time homemaker or being laid off after the index AMI), and (4) unspecified reason for not returning to work (if a patient did not specify a reason for leaving the workforce).

### Patient and Hospital Characteristics

Patient characteristics included baseline demographic characteristics (age, sex, and marital status), socioeconomic status (income and educational level), cardiovascular risk factors (smoking, hypertension, type 2 diabetes, dyslipidemia, and family history of coronary heart disease), history of cardiovascular disease (angina, coronary heart disease, atrial fibrillation, heart failure, history of AMI, history of percutaneous coronary intervention, and history of coronary artery bypass grafting), in-hospital diagnoses (ST-segment elevation or depression, inferior or anterior AMI, Killip class, and left ventricular ejection fraction), in-hospital revascularization procedures (coronary artery bypass grafting or percutaneous coronary intervention), in-hospital complications (recurrent angina, recurrent AMI, atrial fibrillation, cardiopulmonary resuscitation, ventricular tachycardia, ventricular fibrillation, heart failure, infection, stroke, and bleeding), and length of the index hospital stay. Hospital characteristics included hospital location (urban vs rural), type of hospital (tertiary vs others), teaching status (teaching vs nonteaching), coronary artery bypass grafting capacity (yes or no), and percutaneous coronary intervention capacity (yes or no).

### Statistical Analysis

Statistical analysis was conducted from August 9, 2016, to August 15, 2018. We compared patient characteristics between those who returned to work within 12 months after AMI and those who did not return to work using the Mantel-Haenszel test for ordinal variables, χ^2^ test for categorical variables, and Kruskal-Wallis test for continuous variables. We fitted a logistic regression model with the least absolute shrinkage and selection operator method, a shrinkage and variable selection approach for linear regression,^17,18^ to identify patient characteristics associated with return to work at 12 months. To obtain a 95% CI of the coefficient for each selected variable, we conducted a nonparametric bootstrap analysis with 1000 iterations and with replacement.^19,20^ We also fitted a linear multinomial logistic regression model to evaluate the associations between patient characteristics and each of the 4 reasons for not returning to work, with return to work as the reference. To facilitate interpretation of the results, odds ratios of the selected variables were converted to relative risks (RRs) using the method of Zhang and Yu.^21^ All statistical tests were performed using a 2-sided α value of .05. Analyses were conducted using SAS, version 9.4 (SAS Institute Inc). The study followed the guidelines for developing and evaluating a prediction model, described in the Transparent Reporting of a Multivariable Prediction Model for Individual Prognosis or Diagnosis (TRIPOD) statement.^22^ Each of the 22 items of the TRIPOD statement was addressed.

## Results

### Baseline Patient Characteristics

**Figure 1.**
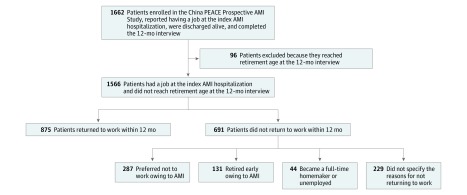
Flowchart of Patient Enrollment AMI indicates acute myocardial infarction; China PEACE Prospective Study of AMI, China Patient-centered Evaluative Assessment of Cardiac Events Prospective Study of Acute Myocardial Infarction.

The final study sample included 1566 patients who were in the workforce before the index hospitalization for AMI and were discharged alive across 53 hospitals (Figure 1). The mean (SD) age of these patients was 52.2 (9.7) years; 169 (10.8%) were aged 65 years or older (Table 1). Among the 1566 patients, 130 (8.3%) were women, 267 (17.0%) had a bachelor’s degree or higher, 550 (35.1%) were agricultural workers, and 1462 (93.4%) were married. The 5 most prevalent comorbidities in the population were hypertension (727 [46.4%]), history of coronary heart disease (620 [39.6%]), dyslipidemia (492 [31.4%]), heart failure (345 [22.0%]), and type 2 diabetes (292 [18.6%]). Most patients (1165 [74.4%]) underwent percutaneous coronary intervention during the hospitalization. The median length of stay was 11.0 days (interquartile range, 8.0-14.0 days). There were no substantial differences in patient baseline characteristics between patients who had a job and those who did not have a job at the time of hospitalization for the index AMI (eTable 1 in the Supplement).

**Table 1.** Comparison of Baseline Characteristics Between Patients Who Did and Patients Who Did Not Return to Work Within 12 Months After Acute Myocardial Infarction

Characteristic	Patients, No. (%)			*P* Value
	Total (N = 1566)	Did Not Return to Work (n = 691)	Returned to Work (n = 875)	
Sociodemographic characteristics				
Age, mean (SD), y	52.2 (9.7)	54.5 (10.1)	50.3 (9.0)	<.001
Female	130 (8.3)	86 (12.4)	44 (5.0)	<.001
Married	1462 (93.4)	642 (92.9)	820 (93.7)	.53
College education	267 (17.0)	90 (13.0)	177 (20.2)	<.001
Occupation				
Worker	395 (25.2)	162 (23.4)	233 (26.6)	<.001
Farmer	550 (35.1)	292 (42.3)	258 (29.5)	
Self-employed	108 (6.9)	43 (6.2)	65 (7.4)	
Other	513 (32.8)	194 (28.1)	319 (36.5)	
Income last year, RMB (US $)				
<30 000 (<4400)	524 (33.5)	249 (36.0)	275 (31.4)	<.001
30 000-70 000 (4400-10 250)	451 (28.8)	182 (26.3)	269 (30.7)	
>70 000 (>10 250)	228 (14.6)	71 (10.3)	157 (17.9)	
Unreported	363 (23.2)	189 (27.4)	174 (19.9)	
Cardiovascular risk factors				
Prior smoking	196 (12.5)	99 (14.3)	97 (11.1)	.05
Hypertension	727 (46.4)	342 (49.5)	385 (44.0)	.03
Type 2 diabetes	292 (18.6)	120 (17.4)	172 (19.7)	.25
Dyslipidemia	492 (31.4)	222 (32.1)	270 (30.9)	.59
Family history of coronary heart disease	187 (11.9)	81 (11.7)	106 (12.1)	.81
Disease history				
Angina	48 (3.1)	25 (3.6)	23 (2.6)	.26
Acute myocardial infarction	80 (5.1)	32 (4.6)	48 (5.5)	.45
Percutaneous coronary intervention	82 (5.2)	35 (5.1)	47 (5.4)	.79
Coronary artery bypass grafting	1 (0.1)	0	1 (0.1)	.37
Coronary heart disease	620 (39.6)	275 (39.8)	345 (39.4)	.88
Ventricular tachycardia or fibrillation	38 (2.4)	17 (2.5)	21 (2.4)	.94
Atrial fibrillation	32 (2.0)	21 (3.0)	11 (1.3)	.01
Heart failure	345 (22.0)	161 (23.3)	184 (21.0)	.28
Valvular heart disease	1 (0.1)	1 (0.1)	0	.26
Peripheral vascular disease	7 (0.4)	3 (0.4)	4 (0.5)	.95
Ischemic stroke	4 (0.3)	3 (0.4)	1 (0.1)	.21
Chronic renal failure	6 (0.4)	3 (0.4)	3 (0.3)	.77
Clinical characteristics				
Non–ST-segment elevation acute myocardial infarction	103 (6.6)	45 (6.5)	58 (6.6)	.93
Killip class 3-4	63 (4.0)	30 (4.3)	33 (3.8)	.57
Inferior wall acute myocardial infarction	627 (40.0)	283 (41.0)	344 (39.3)	.51
Anterior wall acute myocardial infarction	315 (20.1)	121 (17.5)	194 (22.2)	.02
Ischemia >20 min	1166 (74.5)	515 (74.5)	651 (74.4)	.95
Ejection fraction <40%	84 (5.4)	45 (6.5)	39 (4.5)	.07
Coronary artery bypass grafting during index hospitalization	49 (3.1)	27 (3.9)	22 (2.5)	.12
Percutaneous coronary intervention during index hospitalization	1165 (74.4)	523 (75.7)	642 (73.4)	.30
Length of stay, median (IQR), d	11.0 (8.0-14.0)	11.0 (8.0-14.0)	11.0 (8.0-14.0)	.48
In-hospital complications				
Recurrent angina	368 (23.5)	181 (26.2)	187 (21.4)	.03
Recurrent acute myocardial infarction	16 (1.0)	9 (1.3)	7 (0.8)	.33
Atrial fibrillation	27 (1.7)	17 (2.5)	10 (1.1)	.047
Cardiopulmonary resuscitation	17 (1.1)	7 (1.0)	10 (1.1)	.81
Ventricular tachycardia	81 (5.2)	42 (6.1)	39 (4.5)	.15
Ventricular fibrillation	40 (2.6)	14 (2.0)	26 (3.0)	.24
New-onset heart failure	95 (6.1)	47 (6.8)	48 (5.5)	.28
Infection	190 (12.1)	95 (13.7)	95 (10.9)	.08
Stroke	23 (1.5)	16 (2.3)	7 (0.8)	.01
Bleeding	136 (8.7)	67 (9.7)	69 (7.9)	.21
Region				
Central	757 (48.3)	357 (51.7)	400 (45.7)	.02
East	619 (39.5)	265 (38.4)	354 (40.5)	
West	190 (12.1)	69 (10.0)	121 (13.8)	
Urban	1244 (79.4)	555 (80.3)	689 (78.7)	.44
Hospital type				
Teaching hospital	1171 (74.8)	507 (73.4)	664 (75.9)	.26
Tertiary hospital	1285 (82.1)	580 (83.9)	705 (80.6)	.09

Abbbreviations: IQR, interquartile range; RMB, renminbi.

### Returning to Work

In total, 875 patients (55.9%; 95% CI, 53.4%-58.3%) had returned to work within 12 months after the index hospitalization for AMI (Figure 1). Among the 691 patients who did not return, 287 (41.5%) were unable to work and/or preferred not to work because of AMI, 131 (19.0%) retired early because of AMI, 44 (6.4%) became full-time homemakers or were unemployed, and 229 (33.1%) did not specify a reason for not returning to work. We observed differences in baseline patient characteristics by return to work status (Table 1) and reason for not returning to work (Table 2). Compared with patients who returned to work within 12 months, those who did not return were older and more likely to be female, be agricultural workers at the time of the index AMI, have hypertension, and have major adverse events (stroke, atrial fibrillation, and angina) during the index hospital stay. No significant differences were observed in family history of coronary heart disease or length of hospital stay (Table 1). Among patients who did not return to work, the recurrence of angina was the only clinical factor that differed significantly by reason for not returning; there were also differences in sociodemographic factors including age, educational level, income, and occupation (Table 2).

**Table 2.** Baseline Patient Characteristics by Reason for Not Returning to Work

Characteristic	Patients, No. (%)				*P* Value
	AMI-Related (n = 287)	AMI-Related Early Retirement (n = 131)	Full-time Homemaker or Unemployed (n = 44)	Unspecified (n = 229)	
Sociodemographic characteristics					
Age, mean (SD), y	56.1 (9.5)	53.9 (8.4)	56.2 (10.2)	52.6 (11.2)	<.001
Female	38 (13.2)	6 (4.6)	13 (29.5)	29 (12.7)	<.001
Married	265 (92.3)	124 (94.7)	40 (90.9)	213 (93.0)	.80
College education	14 (4.9)	29 (22.1)	3 (6.8)	44 (19.2)	<.001
Occupation					
Worker	68 (23.7)	42 (32.1)	6 (13.6)	46 (20.1)	<.001
Farmer	149 (51.9)	26 (19.8)	26 (59.1)	91 (39.7)	
Self-employed	18 (6.3)	5 (3.8)	4 (9.1)	16 (7.0)	
Other	52 (18.1)	58 (44.3)	8 (18.2)	76 (33.2)	
Income last year, RMB (US $)					
<30 000 (<4400)	133 (46.3)	22 (16.8)	21 (47.7)	73 (31.9)	<.001
30 000-70 000 (4400-10 250)	70 (24.4)	48 (36.6)	11 (25.0)	53 (23.1)	
>70 000 (>10 250)	17 (5.9)	24 (18.3)	2 (4.5)	28 (12.2)	
Unreported	67 (23.3)	37 (28.2)	10 (22.7)	75 (32.8)	
Cardiovascular risk factors					
Prior smoking	48 (16.7)	16 (12.2)	8 (18.2)	27 (11.8)	.31
Family history of coronary heart disease	30 (10.5)	18 (13.7)	7 (15.9)	26 (11.4)	.63
Hypertension	145 (50.5)	62 (47.3)	20 (45.5)	115 (50.2)	.87
Type 2 diabetes	47 (16.4)	32 (24.4)	8 (18.2)	33 (14.4)	.10
Dyslipidemia	94 (32.8)	52 (39.7)	15 (34.1)	61 (26.6)	.08
Disease history					
Angina	13 (4.5)	6 (4.6)	1 (2.3)	5 (2.2)	.46
AMI	15 (5.2)	8 (6.1)	0	9 (3.9)	.35
Percutaneous coronary intervention	13 (4.5)	11 (8.4)	0	11 (4.8)	.13
Coronary artery bypass grafting	0	0	0	0	NA
Coronary heart disease	126 (43.9)	56 (42.7)	14 (31.8)	79 (34.5)	.10
Ventricular tachycardia or fibrillation	8 (2.8)	1 (0.8)	2 (4.5)	6 (2.6)	.47
Atrial fibrillation	10 (3.5)	3 (2.3)	1 (2.3)	7 (3.1)	.91
Heart failure	66 (23.0)	37 (28.2)	8 (18.2)	50 (21.8)	.44
Valvular heart disease	0	1 (0.8)	0	0	.23
Peripheral vascular disease	0	1 (0.8)	0	2 (0.9)	.43
Ischemic stroke	1 (0.3)	1 (0.8)	1 (2.3)	0	.19
Chronic renal failure	1 (0.3)	0	0	2 (0.9)	.61
Clinical characteristics					
Non–ST-segment elevation AMI	19 (6.6)	6 (4.6)	1 (2.3)	19 (8.3)	.35
Killip class 3-4	11 (3.8)	11 (8.4)	2 (4.5)	6 (2.6)	.07
Inferior wall AMI	124 (43.2)	54 (41.2)	19 (43.2)	86 (37.6)	.62
Anterior wall AMI	51 (17.8)	22 (16.8)	12 (27.3)	36 (15.7)	.33
Heart failure on admission	66 (23.0)	37 (28.2)	8 (18.2)	50 (21.8)	.44
Ischemia >20 min	228 (79.4)	96 (73.3)	29 (65.9)	162 (70.7)	.07
Ejection fraction <40%	17 (5.9)	9 (6.9)	2 (4.5)	17 (7.4)	.85
Coronary artery bypass grafting during index hospitalization	11 (3.8)	5 (3.8)	1 (2.3)	10 (4.4)	.93
Percutaneous coronary intervention during index hospitalization	208 (72.5)	106 (80.9)	32 (72.7)	177 (77.3)	.25
Length of stay, median (IQR), d	10.0 (8.0-14.0)	12.0 (8.0-16.0)	10.5 (7.0-15.5)	10.0 (7.0-14.0)	.04
In-hospital complications					
Recurrent angina	92 (32.1)	36 (27.5)	10 (22.7)	43 (18.8)	.008
Recurrent AMI	6 (2.1)	2 (1.5)	1 (2.3)	0	.19
Atrial fibrillation	10 (3.5)	3 (2.3)	1 (2.3)	3 (1.3)	.47
Cardiopulmonary resuscitation	4 (1.4)	1 (0.8)	0	2 (0.9)	.80
Ventricular tachycardia	22 (7.7)	10 (7.6)	2 (4.5)	8 (3.5)	.20
Ventricular fibrillation	5 (1.7)	2 (1.5)	0	7 (3.1)	.49
New-onset heart failure	14 (4.9)	14 (10.7)	2 (4.5)	17 (7.4)	.15
Infection	42 (14.6)	26 (19.8)	6 (13.6)	21 (9.2)	.04
Stroke	11 (3.8)	1 (0.8)	0	4 (1.7)	.13
Bleeding	26 (9.1)	18 (13.7)	5 (11.4)	18 (7.9)	.31
Region					
Central	144 (50.2)	71 (54.2)	22 (50.0)	120 (52.4)	.78
East	112 (39.0)	44 (33.6)	18 (40.9)	91 (39.7)	
West	31 (10.8)	16 (12.2)	4 (9.1)	18 (7.9)	
Urban	203 (70.3)	126 (96.2)	34 (77.3)	192 (83.8)	<.001
Hospital type					
Teaching hospital	193 (67.3)	97 (74.1)	33 (75.0)	184 (80.4)	.01
Tertiary hospital	219 (76.3)	125 (95.4)	34 (77.3)	202 (88.2)	<.001

Abbreviations: AMI, acute myocardial infarction; IQR, interquartile range; NA, not applicable; RMB, renminbi.

### Factors Associated with Returning to Work

**Figure 2.**
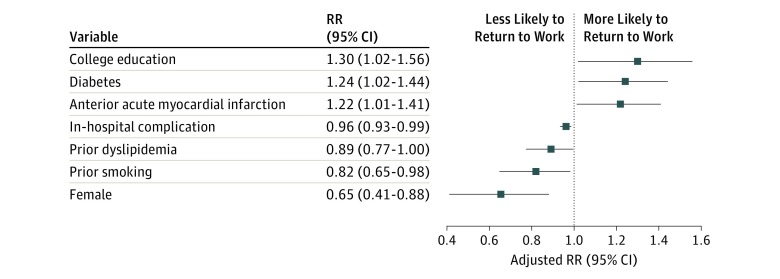
Adjusted Risk Ratio (RR) of Returning to Work Within 12 Months After Discharge for Acute Myocardial Infarction The patient characteristics associated with return to work at 12 months are shown in the first column.

The least absolute shrinkage and selection operator method selected 13 candidate factors that had nonzero coefficients after shrinkage (eTable 2 in the Supplement). The bootstrap simulation removed 6 of these factors with a 95% CI containing zero. The remaining 7 factors included patient demographics (female sex and college degree), comorbidities (type 2 diabetes and dyslipidemia), hospital diagnoses and tests (anterior wall AMI), and in-hospital complications (Figure 2). Factors associated with being more likely to return to work included having a college education (RR, 1.30; 95% CI, 1.02-1.56), history of type 2 diabetes (RR, 1.24; 95% CI, 1.02-1.44), and AMI of the anterior wall (RR, 1.22; 95% CI, 1.01-1.41). Factors associated with a lower likelihood of returning to work included female sex (RR, 0.65; 95% CI, 0.41-0.88), history of smoking (RR, 0.82; 95% CI, 0.65-0.98), history of dyslipidemia (RR, 0.89; 95% CI, 0.77-0.995), and in-hospital complications during the index AMI (RR, 0.96; 95% CI, 0.93-0.99).

The multinomial logistic regression with the least absolute shrinkage and selection operator method and bootstrap analysis identified 15 variables that were statistically significantly associated with at least 1 of the 4 “no return to work” categories relative to the “return to work” category (eFigure in the Supplement). For example, patients who developed adverse events during hospitalization for the index AMI and elderly patients were more likely to not return to work owing to AMI, and female patients were more likely to become full-time homemakers or unemployed after AMI.

## Discussion

In this Chinese national cohort, approximately half of the previously employed individuals returned to work in the year after hospitalization for AMI. With an estimated 2.5 million people in China with a history of AMI,^23^ workforce losses of this magnitude impose a substantial personal and societal burden. Our study identified a diverse array of demographic, socioeconomic, and clinical factors that were associated with return to work and might be amenable to interventions that aim to improve recovery after AMI.

The findings from the China PEACE Prospective Study of AMI extend the prior literature in several ways. Our study provides the most contemporary, nationwide prospective evaluation of the rate of returning to work after AMI in China, as well as the factors associated with returning to work after AMI. Less than 60% of patients in our study returned to work within 12 months after discharge for AMI. This rate is relatively low when compared with data from other countries in which rates have ranged from 74% to 86% during the past 2 decades.^1,4,8,11,24,25^ Several factors may explain this observation. First, the availability of cardiac rehabilitation in China is low, and the quality of rehabilitation services is generally poor.^26^ Cardiac rehabilitation significantly reduces long-term cardiovascular mortality, increases functional capacity, improves levels of pertinent risk factors, and contributes to overall improvement in psychological status and well-being^27,28^; it is also positively associated with a timely return to work.^13^ Second, China has an age-based retirement policy, which currently forces retirement at age 60 years for men and 55 years for women.^29^ Because the mean age of our study population was 52.2 years, enforcement of the retirement policy may have negatively influenced patients’ motivation to return to work, particularly for women who not only have a retirement age 5 years younger than men but also have an older age at onset of AMI compared with men (72.0 vs 65.6 years, respectively).^30^ Long working hours and lack of overtime pay may also have been barriers to returning to work. In 2011, China ranked fourth in the world in terms of hours spent at work,^31^ and overtime is rarely compensated.^32,33^ Last, previous studies have shown that the rate of return to work after AMI varies within and across countries,^8^ with rates of 76% to 86% in the United States,^4,24^ 80% in the United Kingdom,^11^ 76% in the northern Netherlands,^34^ 77% in Iran,^25^ 62% in Germany,^14^ and 74.0% in Finland.^8^ This variation may reflect differences in sampling methods, current unemployment rates, sickness benefits, insurance support for workers, social welfare systems, or employment protection laws across countries.

We identified several sociodemographic and clinical characteristics that were associated with failure to return to work. Contrary to several reports, women in our study were less likely than men to return to work after AMI.^1,8,11,25^ This difference for women could reflect poorer health status, more depression, or less social support after AMI compared with men.^35,36,37^ Even if they did return to work after AMI, more women quit their jobs after returning to work compared with men.^38^ In addition, employed women have less job security, less job control, poorer contractual working conditions, and poorer self-perceived physical and mental health than men, which may negatively affect their decision to return to work.^39^ We also demonstrated that major in-hospital complications were associated with a reduced likehood of returning to work. Although some of these common complications may reflect the inherent nature of the disease,^40^ others are potentially preventable.^41^ The incidence of in-hospital complications has been suggested as a proxy for the quality of hospital care.^42^ Our results suggest that preventing complications during hospitalization might improve overall care and promote return to work after AMI.

More important, we reported the reason for not returning to work from the patients’ perspective. Previous studies showed that patients’ perceptions of illness affected their decisions to return to work after AMI.^14,43^ However, comparison of patients who did not attribute their failure to return to work to the AMI with those who did not return to work because of the AMI revealed no significant differences in cardiovascular risk factors, disease history, or clinical characteristics. Such findings indicate that sociodemographic characteristics, such as age, educational level, occupation, and income, may be more important factors in the decision to return to work.

### Implications

From a societal perspective, our study is of particular importance for China. Our findings signal the need for interventions that promote a timely return to work after AMI in China to mitigate a preventable decline in the working-age population. Labor-intensive industries are still a vital component of China’s economy, and economic growth has relied, at least in part, on the country’s high ratio of working-age to non–working-age individuals. However, China’s working-age population declined from 2013 to 2015,^44^ and the morbidity burden of AMI continues to rise.^23^ The World Bank estimates that the number of people with AMI in China will increase from 2.5 million to 23 million by 2030.^45^ Promoting a return to work after AMI in China may slow the decline in the workforce. The Chinese government’s current consideration of older retirement age may further increase the need to manage this important issue. Our study confirms that factors associated with returning to work after AMI in Western societies are also essential determinants in China (eg, history of smoking).^7,46^ Diabetes and prior dyslipidemia may also be associated with the return to work after AMI, although this finding is contrary to a previous study.^24^ In conclusion, we identified 2 clear targets for intervention after AMI: reducing in-hospital complications and promoting smoking cessation.

### Limitations

Certain limitations should be considered when interpreting our results. Only patients who completed the interview at 12 months were included, possibly introducing selection bias. Only 8.3% of our total study population were women. This low proportion likely reflects a combination of the relatively young age of retirement for women in China (55 years) and the older age at onset of first AMI in women compared with men.^7^ Women may have already retired before experiencing their first AMI. There were more unemployed women in the baseline than employed (324 of 958 [33.8%] vs 130 of 1566 [8.3%], respectively; eTable 1 in the Supplement). Nevertheless, the lack of women may limit the generalizability of the study. We fit fixed-effects models to conduct the analysis. Patients, however, may be clustered within hospitals and regions. We did not have data for the specific date of each patient’s return to work after AMI and the duration that the patient remained at the job. It is possible that patients who returned to work after AMI later withdrew from the workforce before our 12-month assessment. These patients were counted as having not returned to work, and they may have had a better recovery than those who never returned to work at all. We also had no information on other events after hospitalization, such as recurrent AMI, rehospitalization, and bleeding, which may influence the return to work. Although 229 of the 691 patients (33.1%) in our study who failed to return to work declared that they left the workforce for reasons unrelated to AMI, we did not have detailed explanations for these decisions. Such information would be helpful in guiding patient-centered interventions, and close attention to patient decision making is warranted in future studies. We did not collect information on the working environment or patient-reported quality of work factors such as job satisfaction, motivation to resume work, workplace stress, and job security. These factors may influence a person’s decision to reenter the labor force beyond health and socioeconomic considerations.

## Conclusions

Among employed patients hospitalized with AMI in China, nearly half did not return to work within 12 months. Women, prior smokers, and those who experienced adverse in-hospital events were less likely to return to work after AMI. Reducing in-hospital complications, in particular, may be an important target for interventions focused on the timely return to the workforce after AMI.

## Notes

Supplement. **eTable 1.** Comparison of Baseline Characteristics by Employment Status at Baseline Among Patients Who Were Discharged Alive, Did Not Transfer to Another Acute-Care Hospital, Agreed to Participate in the Follow-up Surveys, Completed the 12-Month Interview, and Did Not Reach Retirement Age **eTable 2**. Factors Selected by a Logistic Model With the LASSO Method **eFigure.** Variables Statistically Significantly Associated With at Least 1 of the 4 “No Return to Work” Categories
