# Supplementary material for: Factors Associated With Return to Work After Acute Myocardial Infarction in China
Source: JAMA Netw Open. 2018 Nov 21;1(7):e184831. doi: 10.1001/jamanetworkopen.2018.4831 (PMC6324382; doi:10.1001/jamanetworkopen.2018.4831)
Supplement: Supplement. — eTable 1. Comparison of Baseline Characteristics by Employment Status at Baseline Among Patients Who Were Discharged Alive, Did Not Transfer to Another Acute-Care Hospital, Agreed to Participate in the Follow-up Surveys, Completed the 12-Month Interview, and Did Not Reach Retirement Age eTable 2. Factors Selected by a Logistic Model With the LASSO Method eFigure. Variables Statistically Significantly Associated With at Least 1 of the 4 “No Return to Work” Categories [file jamanetwopen-1-e184831-s001.pdf]

Supplementary Online Content

Jiang Z, Dreyer RP, Spertus JA, et al; China Patient-centered Evaluative Assessment of Cardiac Events (PEACE) Collaborative Group. Factors associated with return to work after acute myocardial infarction in China. *JAMA Netw Open.* 2018;1(7):e184831. doi:10.1001/jamanetworkopen.2018.4831

eTable 1. Comparison of Baseline Characteristics by Employment Status at Baseline Among Patients Who Were Discharged Alive, Did Not Transfer to Another Acute-Care Hospital, Agreed to Participate in the Follow-up Surveys, Completed the 12-Month Interview, and Did Not Reach Retirement Age
eTable 2. Factors Selected by a Logistic Model With the LASSO Method
eFigure. Variables Statistically Significantly Associated With at Least 1 of the 4 "No Return to Work" Categories

This supplementary material has been provided by the authors to give readers additional information about their work.

© 2018 Jiang Z et al. *JAMA Network Open.*

eTable 1. Comparison of Baseline Characteristics by Employment Status at Baseline Among Patients Who Were Discharged Alive, Did Not Transfer to Another Acute-Care Hospital, Agreed to Participate in the Follow-up Surveys, Completed the 12-Month Interview, and Did Not Reach Retirement Age

Characteristics	Total sample (n=2524)	Unemployed (n=958)	Employed (n=1566)	*P* Value
Sociodemographic characteristics, n (%)				
Age, mean (SD), y	55.7 (11)	61.5 (10.5)	52.2 (9.7)	<.001
Female	454 (18)	324 (33.8)	130 (8.3)	<.001
Married	2282 (90.4)	820 (85.6)	1462 (93.4)	<.001
College education	308 (12.2)	41 (4.3)	267 (17.1)	<.001
Region				.001
Central	1155 (45.8)	398 (41.5)	757 (48.3)	
East	1038 (41.1)	419 (43.7)	619 (39.5)	
West	331 (13.1)	141 (14.7)	190 (12.1)	

© 2018 Jiang Z et al. *JAMA Network Open.*

Urban	1973 (78.2)	729 (76.1)	1244 (79.4)	.049
Hospital type				
Teaching hospital	1884 (74.6)	713 (74.4)	1171 (74.8)	.844
Tertiary hospital	2017 (79.9)	732 (76.4)	1285 (82.1)	<.001
Occupation				<.001
Worker	596 (23.6)	201 (21)	395 (25.2)	
Farmer	908 (36)	358 (37.4)	550 (35.1)	
Self-employed	121 (4.8)	13 (1.4)	108 (6.9)	
Other	899 (35.6)	386 (40.3)	513 (32.8)	
Income last year				<.001
<30,000 RMB, n (%)	1026 (40.6)	502 (52.4)	524 (33.5)	
30,000-70,000 RMB, n (%)	627 (24.8)	176 (18.4)	451 (28.8)	
>70,000 RMB, n (%)	270 (10.7)	42 (4.4)	228 (14.6)	
Unreported, n (%)	601 (23.8)	238 (24.8)	363 (23.2)	

© 2018 Jiang Z et al. *JAMA Network Open.*

**Cardiovascular risk factors, n (%)**				
Prior smoking	1713 (67.9)	526 (54.9)	1187 (75.8)	<.001
Hypertension	1298 (51.4)	571 (59.6)	727 (46.4)	<.001
Diabetes mellitus	517 (20.5)	225 (23.5)	292 (18.7)	.004
Dyslipidemia	763 (30.2)	271 (28.3)	492 (31.4)	.097
Family history of coronary heart disease	300 (11.9)	113 (11.8)	187 (11.9)	.913
**Disease history, n (%)**				
Angina	86 (3.4)	38 (4)	48 (3.1)	.226
Acute myocardial infarction	155 (6.1)	75 (7.8)	80 (5.1)	.006
Percutaneous coronary intervention	140 (5.6)	58 (6.1)	82 (5.2)	.384
Coronary artery bypass grafting	2 (0.1)	1 (0.1)	1 (0.1)	.726
Coronary heart disease	1024 (40.6)	404 (42.2)	620 (39.6)	.200
Ventricular tachycardia/fibrillation	60 (2.4)	22 (2.3)	38 (2.4)	.835
Atrial fibrillation	59 (2.3)	27 (2.8)	32 (2)	.211

© 2018 Jiang Z et al. *JAMA Network Open.*

Heart failure	584 (23.1)	239 (25)	345 (22)	.092
Valvular heart disease	2 (0.1)	1 (0.1)	1 (0.1)	.726
Peripheral vascular disease	10 (0.4)	3 (0.3)	7 (0.5)	.603
Ischemic stroke	13 (0.5)	9 (0.9)	4 (0.3)	.020
Chronic renal failure	17 (0.7)	11 (1.2)	6 (0.4)	.023
**Clinical characteristics, n (%)**				
Non-ST-segment elevation acute myocardial infarction	202 (8)	99 (10.3)	103 (6.6)	<.001
Killip class 3-4	106 (4.2)	43 (4.5)	63 (4)	.572
Inferior wall acute myocardial infarction	977 (38.7)	350 (36.5)	627 (40)	.080
Anterior wall acute myocardial infarction	487 (19.3)	172 (18)	315 (20.1)	.182
Ischemia >20 min	1852 (73.4)	686 (71.6)	1166 (74.5)	.116
Ejection fraction <40%	152 (6)	68 (7.1)	84 (5.4)	.076

© 2018 Jiang Z et al. *JAMA Network Open.*

Coronary artery bypass grafting during index hospitalization	100 (4)	51 (5.3)	49 (3.1)	.006
Percutaneous coronary intervention during index hospitalization	1776 (70.4)	611 (63.8)	1165 (74.4)	<.001
Length of stay, median (IQR), d	11 (8-14)	10 (7-14)	11 (8-14)	.015
**In-hospital complications, n (%)**				
Recurrent angina	648 (25.7)	280 (29.2)	368 (23.5)	.001
Recurrent acute myocardial infarction	26 (1)	10 (1)	16 (1)	.957
Atrial fibrillation	62 (2.5)	35 (3.7)	27 (1.7)	.002
Cardiopulmonary resuscitation	29 (1.2)	12 (1.3)	17 (1.1)	.702
Ventricular tachycardia	114 (4.5)	33 (3.4)	81 (5.2)	.043
Ventricular fibrillation	57 (2.3)	17 (1.8)	40 (2.6)	.201
New-onset heart failure	163 (6.5)	68 (7.1)	95 (6.1)	.306
Infection	359 (14.2)	169 (17.6)	190 (12.1)	<.001

© 2018 Jiang Z et al. *JAMA Network Open.*

Stroke	51 (2)	28 (2.9)	23 (1.5)	.012
Bleeding	213 (8.4)	77 (8)	136 (8.7)	.570

IQR, interquartile range; RMB, renminbi; SD, standard deviation.

© 2018 Jiang Z et al. *JAMA Network Open.*

eTable 2. Factors Selected by a Logistic Model With the LASSO Method

**Parameter**	**Estimate**
Female	-0.471875799
Age	-0.201888829
In-hospital complication	-0.169507885
History of dyslipidemia	-0.16748218
History of smoking	-0.228613275
History of hypertension	-0.13858972
Comorbidity score	-0.049881831
Current smoking	-0.140709345
Length of stay	0.009966384
Administration medicine score	0.080598491
Diabetes mellitus	0.268694047
Anterior wall acute myocardial infarction	0.247252728
College education	0.308260281

© 2018 Jiang Z et al. *JAMA Network Open.*

eFigure. Variables Statistically Significantly Associated With at Least 1 of the 4 "No Return to Work" Categories

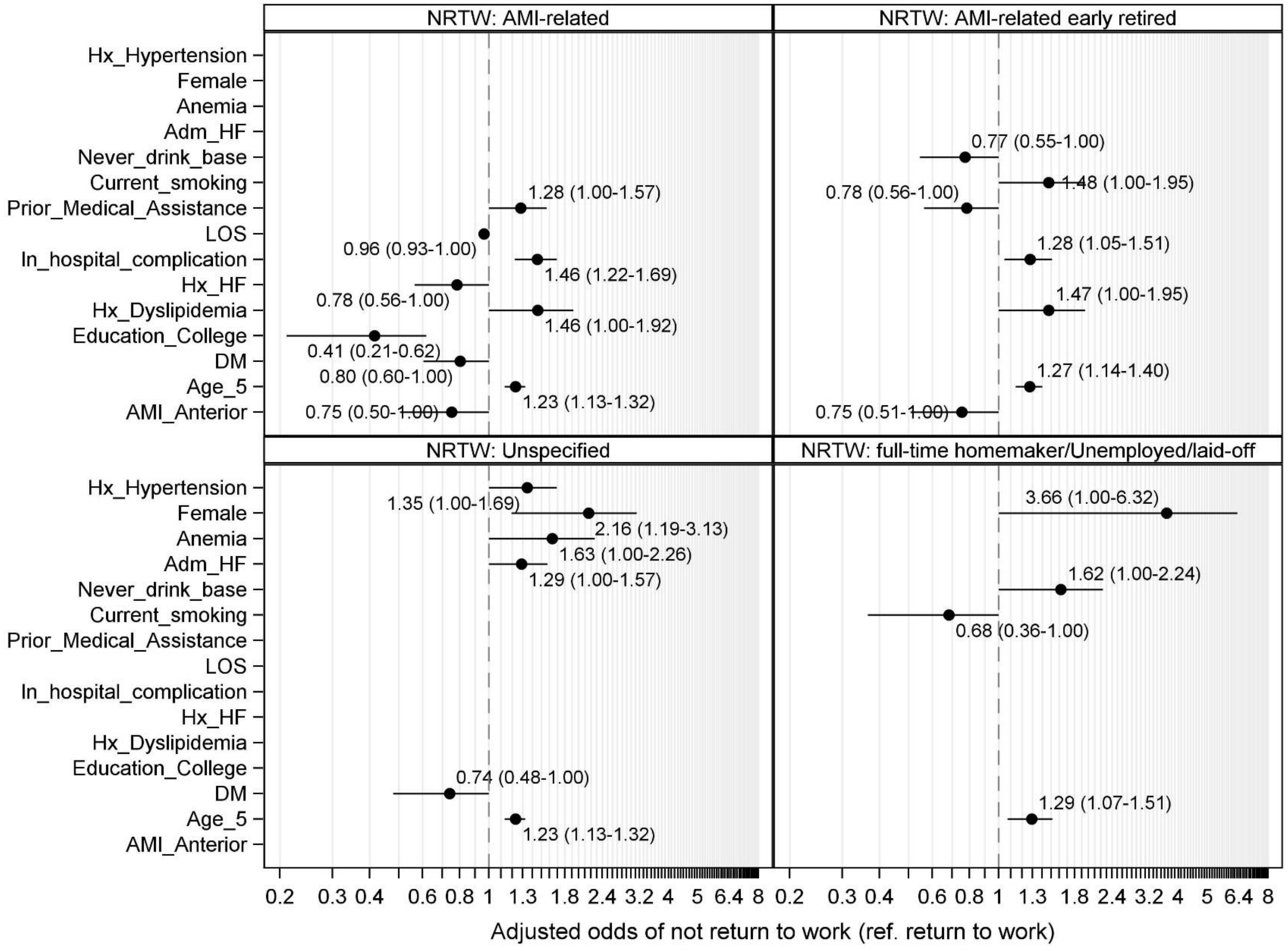

NRTW=Non-return to work

© 2018 Jiang Z et al. *JAMA Network Open.*
